# Supplementary material for: Analysis of big data job requirements based on K-means text clustering in China
Source: PLoS One. 2021 Aug 5;16(8):e0255419. doi: 10.1371/journal.pone.0255419 (PMC8341572; doi:10.1371/journal.pone.0255419)
Supplement: Attachment — Submitted filename: Response to Reviewers.docx [file pone.0255419.s002.docx]

Response to Reviewers

Dear Editors and Reviewers:

Thank you for your letter and for the reviewers’ comments concerning our manuscript entitled “Analysis of Big Data Job Requirements based on K-means Text Clustering in China” (ID: PONE-D-21-07023). Those comments are all valuable and very helpful for revising and improving our paper, as well as the important guiding significance to our researches. We have studied comments carefully and have made correction which we hope meet with approval. Revised portion are marked in red in the paper. The main corrections in the paper and the responds to the reviewer’s comments are as flowing:

Responds to the reviewer’s comments:

Reviewer #1:

1. Response to comment: Make all the data available and clear

Response: Considering the Reviewer’s suggestion, we have added Table 1 on the basis of the original text to show the data we collected, and listed specific examples in 3.1 data collection, so that readers can better understand the content of the data we collected.

2. Response to comment: How the data was preprocessed

Response: We are sorry that we did not show the detailed data preprocessing process. In the revised version, we added Figure 1 to illustrate the specific data processing process in 3.2 data procession.

Special thanks to you for your good comments.

Reviewer #2:

1. Response to comment: The introduction section lacks in problem statement and motivation of the study

Response: We have re-written this part according to the Reviewer’s suggestion. We restate the question and motivation of the study in the introduction.

2. Response to comment: There is immense need to give arguments in favor of conducting this study

Response: It is really true as Reviewer suggested that this article lacks theoretical basis. Therefore, in the new version, we add three references as the theoretical basis and support of the research.

Special thanks to you for your good comments.

We tried our best to improve the manuscript and made some changes in the manuscript. These changes will not influence the content and framework of the paper. And here we did not list the changes but marked in red in revised paper.

We appreciate for Editors/Reviewers’ warm work earnestly, and hope that the correction will meet with approval.

Once again, thank you very much for your comments and suggestions.
